# Single cell profiling of phospho-protein levels in chronic lymphocytic leukemia

**DOI:** 10.18632/oncotarget.23949

**Published:** 2018-01-04

**Authors:** Ida K. Myhrvold, Andrea Cremaschi, Johanne U. Hermansen, Geir E. Tjønnfjord, Ludvig A. Munthe, Kjetil Taskén, Sigrid S. Skånland

**Affiliations:** ^1^ Centre for Molecular Medicine Norway, Nordic EMBL Partnership, University of Oslo and Oslo University Hospital, Oslo, Norway; ^2^ K. G. Jebsen Centre for Inflammation Research, University of Oslo, Oslo, Norway; ^3^ K. G. Jebsen Centre for Cancer Immunotherapy, University of Oslo, Oslo, Norway; ^4^ Oslo Centre for Biostatistics and Epidemiology (OCBE), University of Oslo, Oslo, Norway; ^5^ Department of Haematology, Oslo University Hospital, Oslo, Norway; ^6^ Centre for Immune Regulation, Department of Immunology, University of Oslo, Oslo University Hospital, Oslo, Norway; ^7^ Institute of Clinical Medicine, University of Oslo, Oslo, Norway; ^8^ Department of Infectious Diseases, Oslo University Hospital, Oslo, Norway

**Keywords:** chronic lymphocytic leukemia, phospho-specific flow cytometry, signaling, STAT3

## Abstract

Chronic lymphocytic leukemia (CLL) has a high incidence and a steeply growing prevalence in the Western world. The heterogeneity of the disease necessitates individual mapping of biology and predicted drug response in each patient as basis for administration of tailored treatments. Cell signaling aberrations may serve as biological indicators for suitable therapy. By applying phospho-specific flow cytometry, we mapped basal and induced phosphorylation levels of 20 phospho-epitopes on proteins relevant to B-cell signaling in B cells from 22 CLL patients and 25 normal controls. The signaling response of the cytostatic drugs fludarabine, doxorubicin and vincristine was also investigated. CLL cells exerted similar or lower basal phosphorylation levels compared to normal B cells, with the exception of STAT3 (pY705) which was increased. Interestingly, STAT3 inhibitors normalized the STAT3 (pY705) level and reduced cell viability. Vincristine treatment significantly modulated phosphorylation levels in CLL cells, while no effect was observed in controls or after fludarabine or doxorubicin treatment. After BCR stimulation, CLL cells showed a tendency towards impaired phosphorylation levels, significant for several of the analyzed proteins. However, the level of Akt (pS473) was more potently induced in *IgHV* unmutated CLL (UM-CLL) patient samples and was significantly higher than in M-CLL samples. Importantly, the PI3Kδ inhibitor idelalisib potently reversed the effect of anti-IgM on Akt (pS473). Thus, signaling aberrations could be identified by phosphoflow cytometry and aberrant signaling could be normalized by small molecule drugs. This approach can identify relevant drug targets as well as drug effects in the individual patient.

## INTRODUCTION

Chronic lymphocytic leukemia (CLL) is one of the most prevalent B-cell neoplasias in the Western world [1]. The heterogeneity of the disease results in variable clinical courses with survival ranging from one to more than 15 years [2]. Several molecular and cellular markers have been identified as prognostic markers and can predict disease progression. In particular, immunoglobulin heavy-chain variable gene (IgHV) mutational status, chromosomal abnormalities and expression of CD38 and Zeta-chain-associated protein kinase of 70 kDa (ZAP70) are well established markers [3].

**Figure 5:**
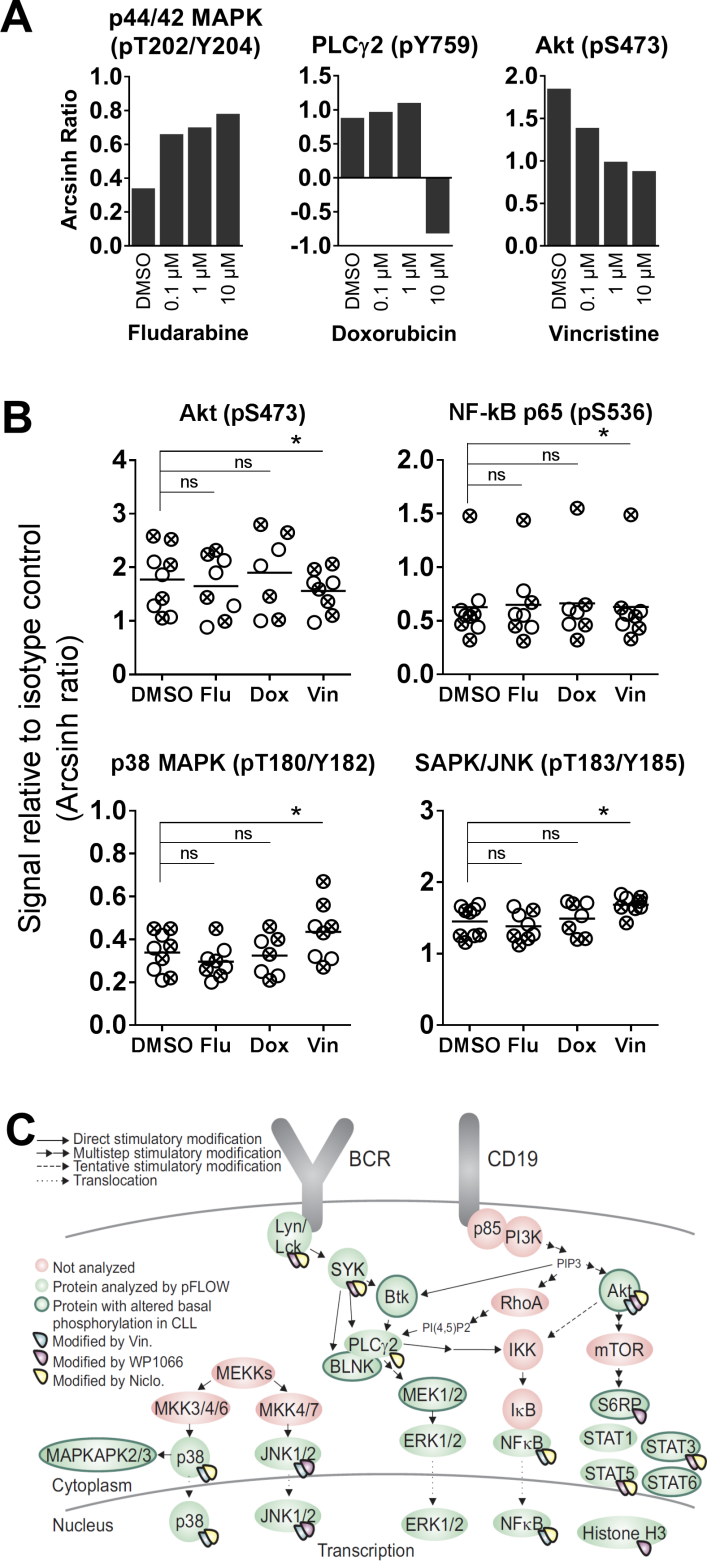
Cytostatic drug-induced phosphorylation levels in CLL B cells (**A**) B cells from healthy donors were incubated with the indicated concentrations of fludarabine, doxorubicin or vincristine for 20 min, before stimulation with anti-IgM for 3 min. The cells were then processed as in Figure 1, and the fluorescence intensity signals were measured relative to unstimulated samples and shown as arcsinh ratio. (**B**) CLL cells were incubated with DMSO (0.001%), fludarabine (Flu; 10 μM), doxorubicin (Dox; 1 μM) or vincristine (Vin; 10 μM) for 20 min. The cells were then processed and analyzed as described in Figure 1. The DMSO data are from Figure 1A. Indicated *p*-values were calculated by multiple comparison testing with Holm-Sidak’s correction (^*^ *p* < 0.05, ns: not significant). Only phospho-proteins where at least one of the drugs induced significant changes are shown. Symbols with a cross represent UM-CLL samples, while open circles represent M-CLL samples. Horizontal bars indicate calculated mean. (**C**) Illustration highlighting proteins that show significantly altered basal or drug-induced phosphorylation levels in CLL B cells relative to normal controls.

The B cell receptor (BCR) pathway with its associated signaling proteins is essential for normal immune function and for survival and proliferation of B cells. The mutational status of the IgHV is a strong predictor for disease outcome in CLL, suggesting that signaling through the BCR plays an important role in CLL pathogenesis [4]. The BCR is composed of covalently linked immunoglobulin heavy and light chains and is tightly associated with the membrane integrated CD79a and b. After antigen stimulation, the BCR aggregates, and the CD79a/b propagate an activation signal to a Sarcoma (Src)-family protein tyrosine kinase, normally Lyn (see Figure 5C for a simplified cartoon of BCR signaling), which then induces phosphorylation of the immunoreceptor tyrosine-based activation motifs (ITAMs) on CD79a and b. The phosphorylated ITAMs serve as docking sites for SH2-domain containing proteins, most often Spleen tyrosine kinase (SYK). There is some redundancy in signaling, ZAP70, which is highly expressed in CLL cells with an aggressive course, may also contribute [5]. The signaling continues with formation of the BCR signalosome and the recruitment of B cell linker protein (BLNK) to CD79b. BLNK serves as a docking site for Bruton’s tyrosine kinase (Btk), Phospholipase Cγ2 (PLCγ2) and the adaptor protein Growth factor receptor-bound protein 2 (GRB2). This BCR signalosome generates a wide variety of downstream effects, including activation of the PI3K-Akt-mTOR pathway and the Ras-Raf-MEK-ERK pathway [6].

After assembly of the BCR signalosome, signaling through GRB2, the Son of sevenless (SOS) and rat sarcoma protein (Ras) is propagated downstream leading to activation of the Raf proto-oncogene serine/threonine-protein kinase (Raf), followed by Mitogen activated protein kinase kinase (MEK), and Mitogen activated protein kinase (p44/42 MAPK/ERK1/2). This Ras-Raf-MEK-ERK pathway regulates the expression of the Activator protein 1 (AP1) which is a transcription factor important for proliferation and differentiation [6].

The PI3K-Akt-mTOR pathway is involved in many cellular functions, including cell cycle progression, cell survival and apoptosis. It is one of the most commonly mutated pathways in cancer, and increased activity of this pathway has been observed in many malignancies, including leukemias [4]. After BCR activation, the PI3K converts phosphatidylinositol-4,5-bisphosphate (PIP2) to phosphatidylinositol-3,4,5-trisphosphate (PIP3), which serves as a membrane docking site for the plextrin homology (PH) domains in Akt and Phosphoinositide-dependent protein kinase 1 (PDK1). Mammalian target of rapamycin (mTOR) is recruited, and both mTOR and PDK1 phosphorylate Akt. Fully activated Akt phosphorylates various target proteins, leading to inhibition of apoptosis and promotion of cell survival [4].

A central signaling pathway in CLL is the JAK/STAT pathway [7]. Activation of this pathway stimulates cell migration, proliferation, differentiation and apoptosis which are crucial for growth and development of the immune system [8]. When a ligand, such as a growth hormone or a cytokine like Interferon γ (IFNγ), binds to its cognate receptor, a receptor dimer is formed and Janus kinase (JAK) tyrosine kinases are recruited. The JAKs phosphorylate additional targets, including the STATs. The STATs are latent transcription factors ready to activate or repress transcription of target genes, including CD38 [7, 8].

Here, basal and induced signaling in CLL cells relative to normal controls were analyzed by phosphoflow cytometry in order to map signaling aberrations that can provide indications for targeted therapy. Furthermore, the signaling responses of the purine analogue fludarabine, the vinca alkaloid vincristine and the anthracycline doxorubicin were characterized. These cytostatic drugs are currently in use for the treatment of CLL [9]. However, their effects on signaling responses have to our knowledge not previously been characterized in detail. The present study suggests that phosphoflow cytometry has the potential to identify relevant drug targets as well as drug effects in the individual patient.

## RESULTS

### Characterization of basal phosphorylation levels in CLL and normal B cells

In order to identify signaling aberrations in CLL cells relative to normal B cells, we investigated both basal and induced phosphorylation levels of 20 different phospho-epitopes on signaling proteins relevant for the BCR signaling pathway.

**Figure 1:**
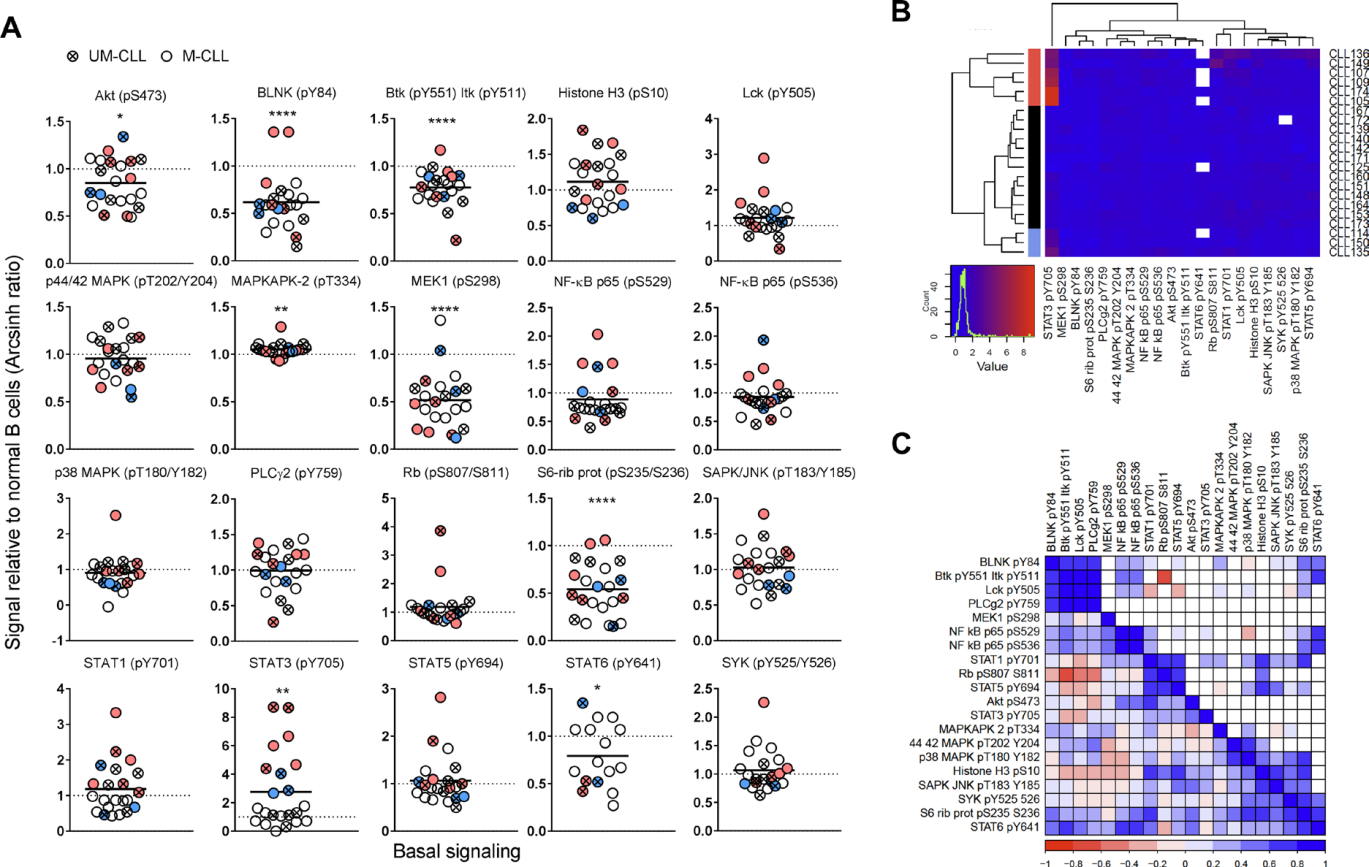
Basal phospho-protein levels in CLL B cells (**A**) Unstimulated B cells from healthy donors (*n* = 25) and CLL patients (*n* = 22) were fixed, permeabilized and stained with anti-CD19 surface marker and indicated phospho-protein specific antibodies as described in Materials and Methods. The fluorescence signals were detected by flow cytometry and the data were analyzed in Cytobank. The basal fluorescence intensity signals were calculated relative to IgGk isotype control and shown as arcsinh ratio. The relative phospho-protein levels in CLL B cells were normalized to normal controls. Significant *p*-values are indicated (^*^p < 0.05, ^**^p < 0.01, ^***^p < 0.001, ^****^p < 0.0001), and were calculated by unpaired two-samples *t*-test. Symbols with a cross represent UM-CLL patient samples, while open circles represent M-CLL patient samples. Pink and blue symbols refer to STAT3 (pY705) high samples which cluster together (see B). Horizontal bars indicate calculated mean. (**B**) Phospho-proteins and CLL samples shown in (A) were grouped via hierarchical agglomerative clustering (linkage method used was Ward’s method). Missing data points are shown in white. Pink and blue clusters indicate patient samples with high level of STAT3 (pY705) (see A). (**C**) Matrix comparing paired phospho-protein sample correlations in CLL samples (lower left triangle, *n* = 22). Higher correlation is indicated by darker colour (red for negative, blue for positive). Upper right triangle shows only those entries associated with correlations significantly different from 0 (assessed by using Pearson’s r-test, *p*-value < 0.05).

**Figure 4:**
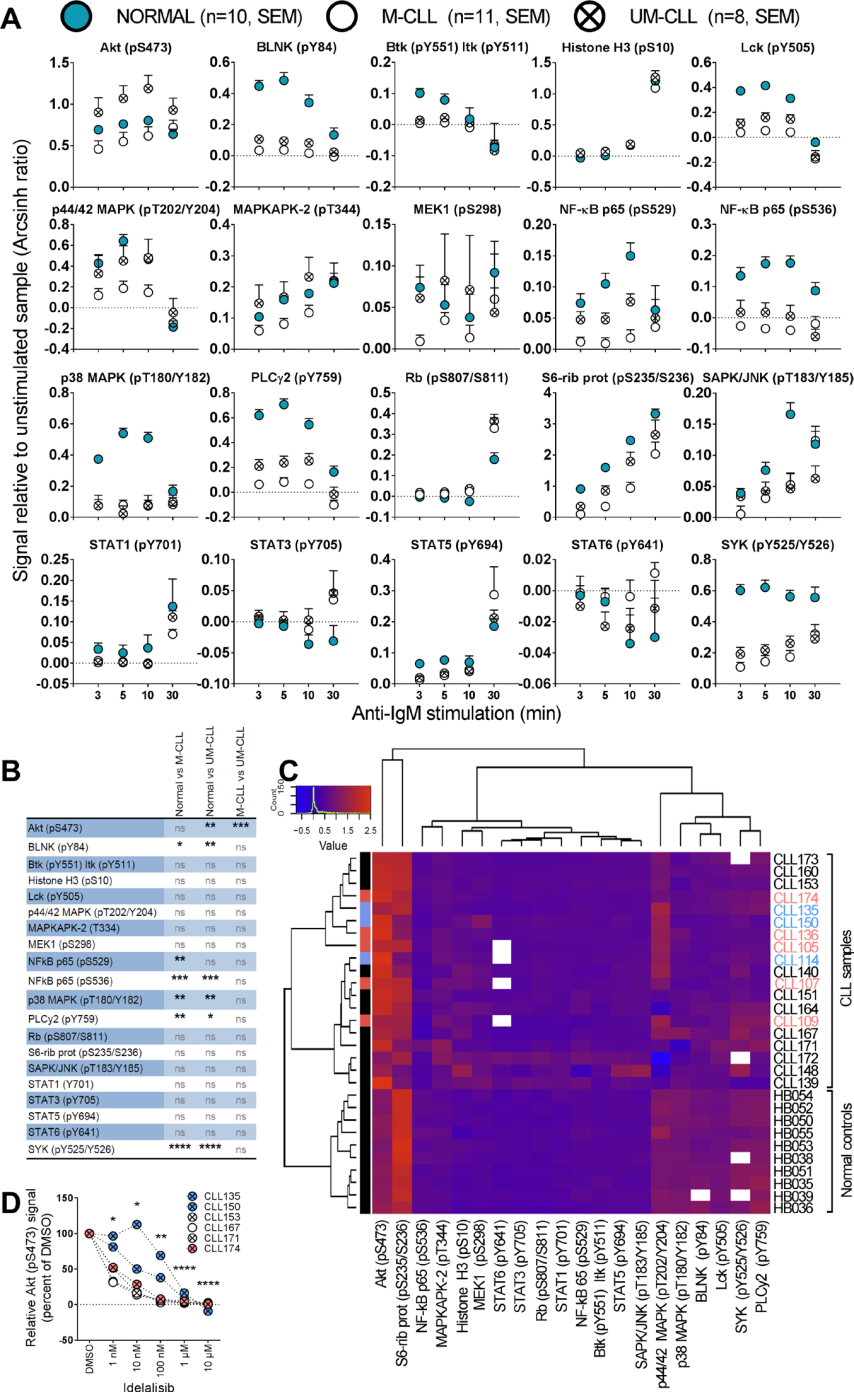
Anti-IgM induced signaling in B cells from healthy donors and CLL patients (**A**) B cells from healthy donors (mean + SEM, *n* = 10, filled circles) or CLL patients (mean + SEM, *n* = 11 (M-CLL, open circles) and *n* = 8 (UM-CLL, crossed circles)) were stimulated with anti-IgM for the specified time period, then processed and analyzed as described in Figure 1. The fluorescence intensity signals were measured relative to unstimulated samples and shown as arcsinh ratio. (**B**) Summary of multiple comparison testing with Holm-Sidak’s correction for normal, M-CLL and UM-CLL groups as shown in (A). Asterisks indicate significant *p*-value (^*^p < 0.05, ^**^p < 0.01, ^***^ *p* < 0.001, ^****^ *p* < 0.0001), ns: not significant. (**C**) Phospho-proteins and B-cell samples shown in (A) were grouped via hierarchical agglomerative clustering (linkage method used was Ward’s method), analogously to Figure 1B. The heatmap shows signals induced by anti-IgM stimulation for 5 min. Missing data are indicated in white. Patients highlighted in pink and blue refer to the STAT3 (pY705) high patients as shown in Figure 1. (**D**) B cells were incubated with DMSO or the indicated idelalisib concentration for 20 min before anti-IgM stimulation for 3 min. The cells were then fixed, barcoded and permeabilized before antibody staining and analysis as described in Materials and Methods. *P* values were calculated by multiple comparison testing with Holm-Sidak’s correction. Asterisks indicate significant *p*-value (^*^p < 0.05, ^**^p < 0.01, ^***^p < 0.001, ^****^p < 0.0001).

When the phospho-protein levels in 22 CLL samples were analyzed relative to the mean of normal controls, the basal phosphorylation levels were shown to be reduced and significantly different (p ϵ 0.0001-0.05) for the phospho-proteins BLNK (pY84), Btk (pY551) & Itk (pY511), MEK1 (pS298), S6-Ribosomal protein (pS235/236) and STAT6 (pY641). MAPKAPK-2 (pT334) and STAT3 (pY705) were increased and statistically different from controls (*p* < 0.001) (Figure 1A). Nine patient samples, including both UM-CLL and M-CLL type, showed more than two-fold increase in STAT3 (pY705) level relative to controls. By using an agglomerative hierarchical clustering procedure (Euclidean distance – Ward’s linkage method) on the CLL samples, the STAT3 (pY705)-high samples were grouped into two distinct clusters (indicated in pink and blue in Figure 1B). The two clusters suggest similar signaling patterns among the patients, with the larger group being characterized by higher STAT3 (pY705) levels (pink in Figure 1A and 1B. See also Figure 4C). Supplementary Figure 1 shows the non-normalized phospho-protein levels in both CLL samples and normal controls. Unpaired two-samples *t*-test was used to assess the difference between the CLL and normal samples, with *p* values corrected for multiple comparisons using Holm-Sidak’s method (Supplementary Figure 1A). In Supplementary Figure 1B, data for 18 out of the 20 non-normalized phospho-proteins were used to perform an agglomerative hierarchical clustering (Euclidean distance – average linkage method) of both CLL and normal B cells (*n* = 10, see Figure 4C). For this analysis, STAT6 (pY641) was removed due to the presence of several missing values, as well as MAPKAPK-2 (pT334) since its high values reduced the visibility of the variability of the data. Log-transformation was then applied to further emphasize the differences in the signals. In this analysis, several of the STAT3 (pY705)-high samples still grouped together, but not to the same extent as observed in Figure 1B (Supplementary Figure 1B).

In Figure 1C, the correlation between the phospho-proteins in unstimulated CLL samples was investigated. The correlation coefficient ranges from −1 (dark red) to +1 (dark blue). The diagonal of the matrix separates the lower left triangle which shows the sample correlations for all phospho-protein pairs from the upper right triangle which shows the significant correlations only (*p*-value < 0.05) (Figure 1C). As expected, the two phospho-epitopes on NF-κB p65 (pS529 and pS536) displayed a positive correlation of approximately +1. Furthermore, the proteins BLNK (pY84), Btk (pY551) Itk (pY511), and PLCγ2 (pY759), which are part of the BCR signalosome, also showed positive correlation (Figure 1C). STAT3 (pY705) showed significant positive correlation only with STAT1 (pY701) and NF-κB p65 (pS529). The correlation patterns could also be visualized in Figure 1A. For example, the majority of the STAT3 (pY705) high samples showed high levels of STAT1 (pY701) and NF-κB p65 (pS529). In agreement with the correlation data, STAT3 signaling has been reported to be highly interconnected with NF-κB signaling [10].

Briefly, aberrant levels of protein phosphorylation were identified in unstimulated CLL cells and a group of samples showed elevated STAT3 (pY705) as well as a similar signaling pattern overall.

### STAT3 inhibitors normalize aberrant STAT3 signaling and reduce cell viability in CLL

**Figure 2:**
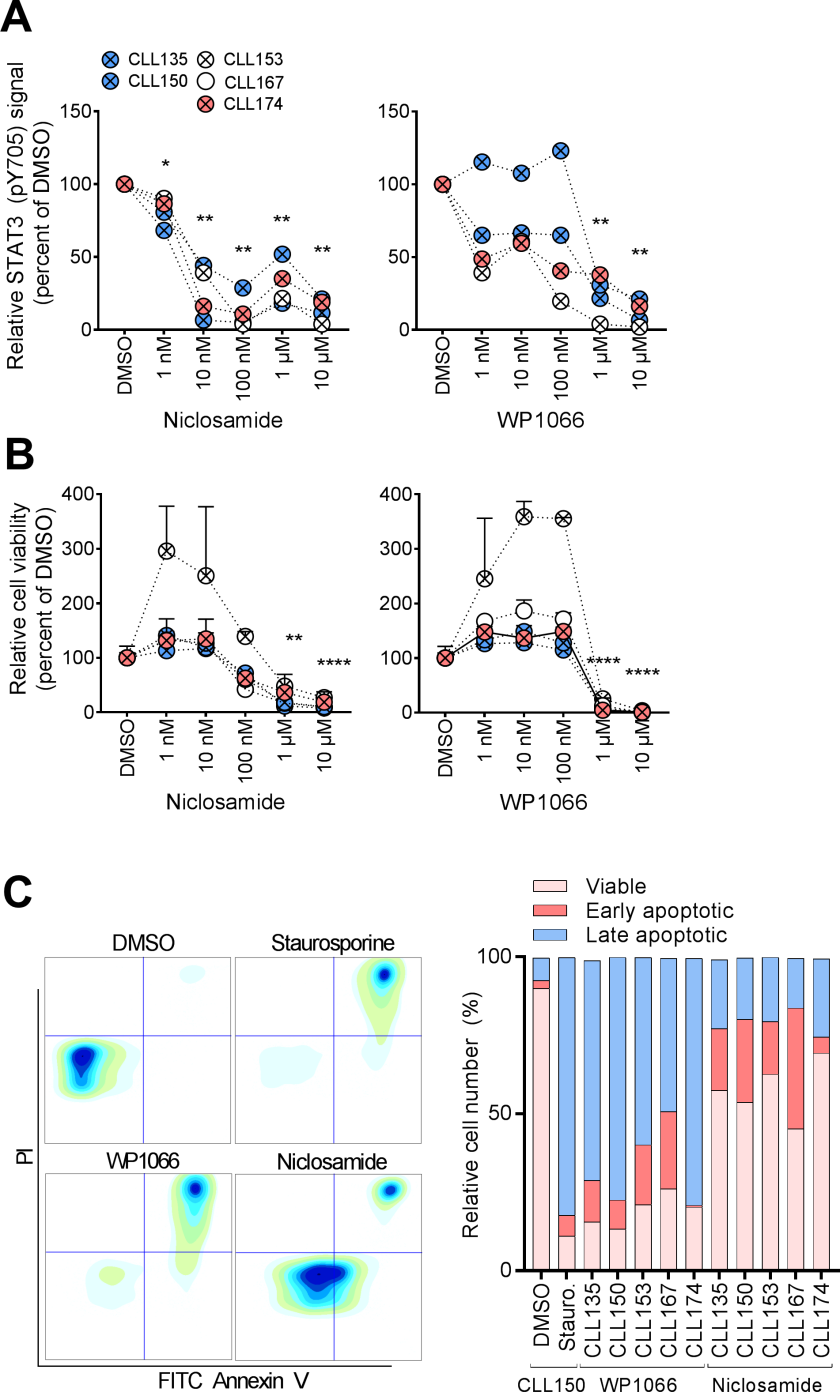
STAT3 inhibitors normalize aberrant CLL signaling (**A**) B cells were incubated with DMSO or the indicated drug concentration for 24 hours, then fixed, barcoded and permeabilized before antibody staining and analysis as described in Materials and Methods. (**B**) CLL cells were co-cultured with CD40L+, BAFF+ and APRIL+ (ratio 1:1:1) L cells for 24 hours prior to initiation of the experiment to prevent induction of spontaneous apoptosis. The L cells were removed, and the CLL cells were incubated with the indicated drugs for 48 hours. CellTiter-Glo reagent was subsequently added and luminescence was recorded after 10 min incubation at room temperature using an EnVision 2102 Multilabel Reader. *N* = 3, SEM. *P* values were calculated by multiple comparison testing with Holm-Sidak’s correction. Asterisks indicate significant *p*-value (^*^p < 0.05, ^**^p < 0.01, ^***^p < 0.001, ^****^p < 0.0001). (**C**) CLL cells were cultured and exposed to drug (10 μM) as described above. Cells treated with staurosporine (1 μM) were included as a positive control. The cells were then stained with FITC Annexin V and PI, and analyzed by flow cytometry. Plots from one patient sample (CLL150) are shown (left). Relative numbers of viable cells (lower left gate), early apoptotic cells (lower right gate) and late apoptotic cells (upper right gate) are plotted for the indicated patient samples (graph, right).

Of clinical significance, STAT3 has been reported to be constitutively active in a large number of cancers including haematological malignancies. However, this characteristic has not previously been shown to comprise CLL [11]. Due to its therapeutic significance, STAT3 is the target in numerous drug discovery research efforts. As shown in Figure 2, the STAT3 inhibitors niclosamide and WP1066 significantly reduced both STAT3 (pY705) levels (Figure 2A) and cell viability (Figure 2B, 2C) of CLL cells in a concentration-dependent manner. Of notice, the ATP-based assay CellTiter-Glo detected drug effects at lower drug concentrations than the Annexin V/PI based assay (Figure 2B, C and data not shown), suggesting that the STAT3 inhibitors reduce cellular metabolism before cell death is induced. These findings indicate that STAT3 signaling is implicated in CLL cell viability, and that the protein may represent a potential therapeutic target. However, additional studies are required in order to conclude whether phospho-flow profiling can predict the response to STAT3 inhibitors.

### Phospho-flow profile of STAT3 inhibitors reveal multiple signaling effects

**Figure 3:**
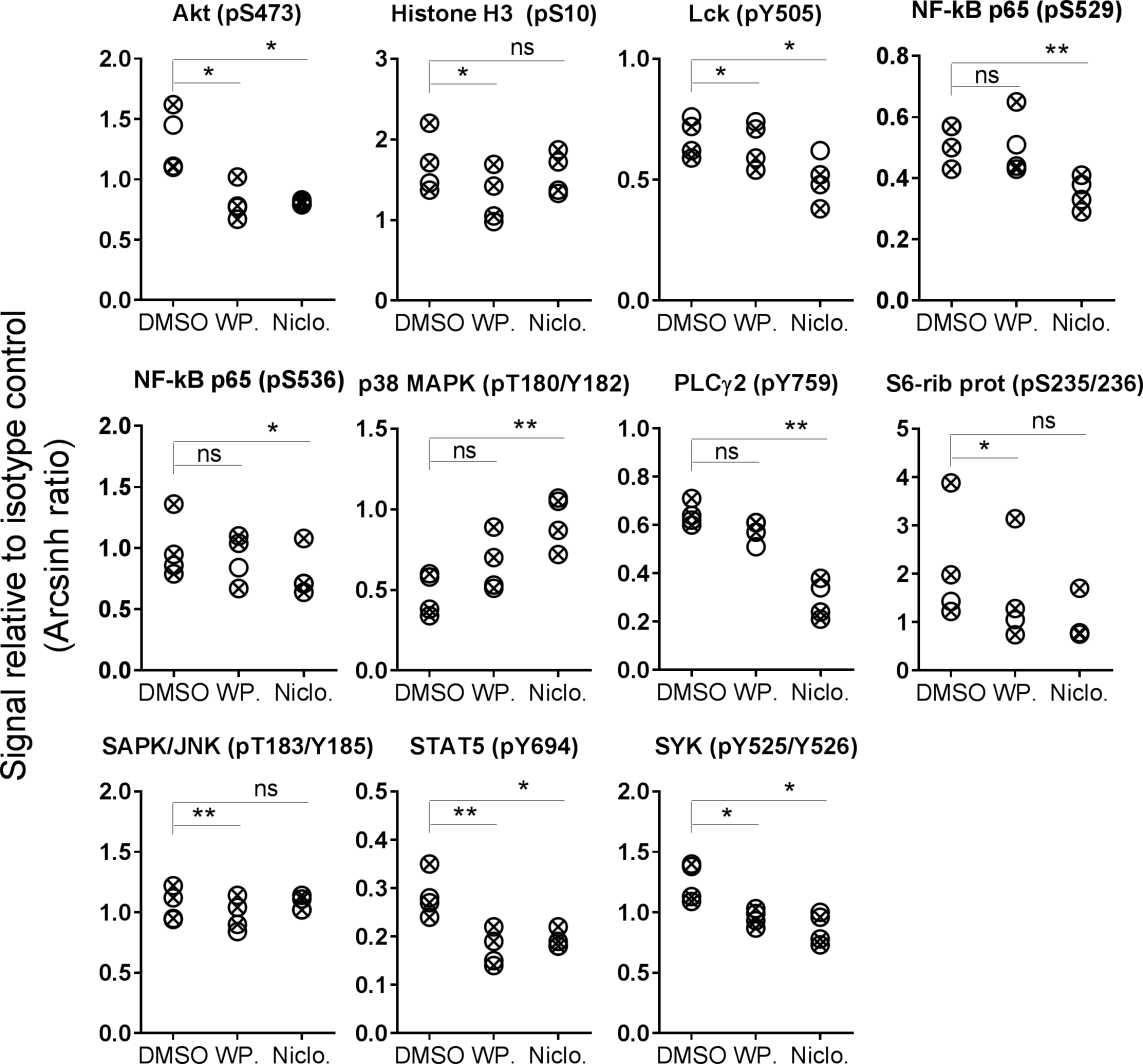
STAT3 inhibitor-induced phosphorylation levels in CLL B cells CLL cells were incubated with DMSO (0.001%), WP1066 (WP.; 10 μM) or niclosamide (Niclo.; 10 μM) for 20 min. The cells were then processed and analyzed as described in Figure 1. Indicated *p*-values were calculated by multiple comparison testing with Holm-Sidak’s correction (^*^p < 0.05, ^**^p < 0.01, ns: not significant). Only phospho-proteins where at least one of the drugs induced significant changes are shown. Symbols with a cross represent UM-CLL samples, while open circles represent M-CLL samples.

In addition to the observed and expected inhibition of STAT3 phosphorylation, a full phospho-flow profiling was carried out using the two STAT3 inhibitors. CLL cells were exposed to a concentration range (ten-fold steps from 1 nM to 10 μM) of niclosamide or WP1066 for 20 min before signaling patterns were analyzed. The results using 10 μM of each drug is shown in Figure 3. Both STAT3 inhibitors induced a significant reduction in Akt (pS473), Lck (pY505), STAT5 (pY694) and SYK (pY525/Y526). Furthermore, both drugs caused additional specific signaling deviations (Figures 3 and 5C). Results suggest that STAT3 inhibitors alter the phosphorylation status of multiple signaling molecules.

### Distinct BCR signaling patterns in CLL and normal B cells

To identify any signaling differences induced through the BCR pathway in CLL cells relative to normal B cells, the cells were next stimulated with anti-IgM and followed for 30 min (Figure 4). It has previously been reported that UM-CLL cells show an increased sensitivity towards anti-IgM stimulation [12]. We observed that UM-CLL cells exhibited a tendency towards higher phospho-protein levels relative to M-CLL cells for the majority of the analyzed proteins, but the effect was statistically significant for Akt (pS473) only (Figure 4A, 4B). For this parameter the phosphorylation-levels in UM-CLL cells also significantly exceeded those detected in normal controls (Figure 4A and 4B). In general, however, the CLL cells were hyporesponsive or showed only minor deviations from normal B cells (Figure 4A and 4B).

The exact cellular origin for CLL cells is still under debate [13], a possible confounding influence in the current results was the choice of normal control cells. These were predominately CD19^+^ B cells that lacked expression of CD5, a differentiation or activation marker of B cells. CLL cells are CD5^+^CD19^+^ cells, while the normal control cells were nearly all CD5^−^CD19^+^. An additional control was therefore included in which signaling patterns were compared between normal CD5^−^CD19^+^ and CD5^+^CD19^+^ cells. As shown in Supplementary Figure 2, no significant differences were observed between these subsets.

In order to investigate whether the signaling patterns in CLL samples could be separated from normal controls, an unsupervised cluster analysis based on phospho-epitope phosphorylation status after 5 min of anti-IgM stimulation was performed. Interestingly, the cluster analysis revealed that samples from healthy donors and CLL patients made up three separate clusters (Figure 4C). Two sub-clusters were observed for the CLL patients, but IgHV mutational status or other patient characteristics did not explain this separation. Interestingly, the STAT3 (pY705) high patient samples to some extent clustered together (Figure 4C). For the phospho-proteins examined, three sub-clusters were identified. One sub-cluster consisted of Akt (pS473) and S6-Ribosomal protein (pS235/S236). These two phospho-proteins showed distinct patterns in CLL samples relative to normal controls, most obviously so for Akt (pS473) which was higher in the CLL cells (Figure 4C). A second sub-cluster consisted of p44/42 MAPK (pT202/Y204), p38 MAPK (pT180/Y182), BLNK (pY84), Lck (pY505), SYK (pY525/Y526) and PLCγ2 (pY759). These phospho-proteins were all low in CLL samples and most of them were statistically different from the levels in normal controls (Figure 4C, 4B). The proteins in the remaining sub-cluster displayed similar levels among all donors (Figure 4C).

In order to investigate whether the aberrantly induced signaling observed in CLL cells could be reversed to normal levels, anti-IgM induced Akt (pS473) signaling was targeted using a range of concentrations of the PI3Kδ inhibitor idelalisib (Figure 4D). This drug is currently in use for treatment of CLL. All patient samples showed reduced Akt (pS473) levels after treatment with increasing concentrations of the drug (Figure 4D), demonstrating that pathway inhibitors can be applied to normalize aberrant signaling in CLL cells.

### Effects of cytostatic drugs on B cell signaling

The effects of the cytostatic drugs fludarabine, doxorubicin and vincristine on B cell signaling were investigated. These drugs are currently used in CLL therapy, but a detailed characterization of their effects on signaling in B cells has to our knowledge not previously been carried out. We chose to study the signaling effects induced by short-time (20 min) incubation with the drugs. In order to determine the optimal working concentrations of these drugs, normal B cells were treated with three different concentrations (0,1 μM, 1 μM and 10 μM) of the drugs, followed by anti-IgM stimulation (Figure 5A). The cells responded in a concentration-dependent manner to the drugs with the most pronounced effect at the highest concentration. However, doxorubicin appeared to be toxic to the B cells at 10 μM (Figure 5A, and data not shown). Based on these results, we chose the working concentrations 10 μM, 1 μM and 10 μM for fludarabine, doxorubicin and vincristine, respectively, which is in line with previous *in vitro* studies [14–16].

No significant changes in phosphorylation levels could be induced by any of the three drugs in B cells from healthy donors (data not shown). However, in CLL cells, some of the 20 investigated phospho-proteins were significantly modulated upon vincristine treatment, but not by fludarabine or doxorubicin (Figure 5B). The observed modifications included lowered Akt (pS473) and NF-κB 65 (p536) signals, and increased p38 MAPK (pT180/Y183) and SAPK/JNK (pT183/Y185) phosphorylation. However, although the modulations were statistically significant, the biological significance of these signaling effects need to be further investigated. It has previously been reported that vincristine induces SAPK/JNK activation in BR ovarian carcinoma cells and MCF-7 breast cancer cells [17]. No significant changes in phosphorylation levels were detected in cells treated with a cytostatic drug followed by anti-IgM stimulation (data not shown). A summary of the proteins which showed altered basal or drug-induced phosphorylation in CLL cells is shown in Figure 5C. All drugs that induced statistically significant signaling alterations are shown.

## DISCUSSION

In the present study, basal and anti-IgM induced phospho-protein levels were characterized in B cells from healthy donors and CLL patients. The phosphorylation levels of 20 different phospho-epitopes on proteins downstream of the BCR were used as read-out in phospho-flow assays. In addition, the effects on signaling responses of the therapeutic cytostatic drugs fludarabine, doxorubicin and vincristine which are commonly used in CLL treatment were investigated. Here, phospho-flow was the method of choice to study CLL signaling. Other techniques such as protein array and reverse phase protein array (RPPA) may be used to quantify the expression levels of phospho-proteins in a medium to high-throughput manner. An important advantage of phospho-flow is that it is based on very versatile flow cytometry analysis that allows for characterization of single cell phenotype as well as detection of inter-cellular heterogeneity. In the present study, the multiplexing of the phospho-flow analysis allowed us to identify aberrations even in this early explorative phase with modest sample size. Our results suggest that more extensive future studies may define multiple targets in the heterogeneous CLL patient population.

Basal signaling in CLL was first investigated, and when the CLL patient samples were analyzed as one population, several deviations were detected relative to the normal control group. Most of these deviations showed lower basal signals in CLL cells, probably as a result of feedback inhibition. However, a significant increase in the basal level of STAT3 (pY705) was observed in about a third of the samples and both in UM-CLL and M-CLL samples. Constitutive serine phosphorylation of STAT1 and STAT3 has been reported in CLL cells, while tyrosine phosphorylation of these STAT proteins was not detected [18]. A recent study on signaling profiles in non-Hodgkin lymphomas suggested that basal STAT3 (pY705) levels were elevated in CLL cells relative to normal B cells, but the statistical significance was not indicated [19]. For other haematological malignancies, constitutive activation of STAT3 has been demonstrated [11]. Cancer cells with constitutive STAT3 activation have been reported to have elevated levels of cell cycle regulating and anti-apoptotic proteins, leading to resistance to apoptosis [11]. Importantly, the two experimental STAT3 inhibitors WP1066 and niclosamide reversed the elevated phosphorylation of STAT3 and reduced the viability of CLL cells. These results suggest that aberrant signaling can be targeted and modulated by drugs that specifically perturb signaling pathways to the extent that they may be applicable for future clinical testing.

A previous study on signaling in SLL/CLL cells [20] reported significantly higher basal phosphorylation levels of PLCγ2 (pY759), p44/42 MAPK (pT202/Y204), p38 MAPK (pT180/Y182), NF-κB p65 (pS529), STAT5 (pY694) and STAT6 (pY641) in these cells compared to normal B cells. In the present study, similar elevations in phosphorylation were not observed. Rather, a tendency towards lower basal phosphorylation levels in CLL cells was found, although outliers were present. The inconsistence between the two studies may possibly be due to different experimental approaches. Herein samples from CLL patients were investigated, whereas Blix et al. included samples from both CLL and small lymphocytic lymphoma (SLL) patients [20]. Although these diseases are considered to be similar, SLL B cells have a smaller leukemic subpopulation and reside more strictly at nodal sites [21]. Furthermore, the samples in the present study were obtained from blood whereas Blix et al. prepared single-cell suspensions from collected tumor biopsies [20].

When the effects of the cytostatic drugs fludarabine, doxorubicin and vincristine on cell signaling were investigated, a few significant signaling modulations were observed after vincristine treatment of CLL cells, while no effects were detected in normal B cells or after fludarabine or doxorubicin treatment. SAPK/JNK (pT183/Y185) phosphorylation was significantly increased in CLL patient samples after vincristine treatment. This finding is in line with a previous report where the effect of vincristine was examined in BR ovarian carcinoma cells and MCF-7 breast cancer cells [17]. Wang et al. reported that activation of SAPK/JNK increased in a dose-dependent manner after treatment with vincristine.

BCR signaling, induced after stimulation with anti-IgM for up to 30 minutes, was also characterized. Only Akt (pS473) showed higher phosphorylation-levels in UM-CLL cells relative to both M-CLL and normal B cells. The remaining significant differences indicated lower signaling amplitudes in CLL cells relative to normal B cells. Our observation of hypo-phosphorylation after BCR stimulation was in agreement with previous reports [19, 20].

In summary, basal and anti-IgM induced signaling was characterized in detail in B cells from healthy donors and CLL patients. Signaling aberrations in CLL cells may be used to guide targeted therapy. We recently showed that sCD40L stimulation more efficiently induced phosphorylation of SYK in CLL cells relative to normal B cells, and that SYK inhibitors reduced CD40L-induced proliferation of CLL cells specifically [22]. In the present study we showed that constitutive as well as induced signaling could be reversed by the use of specific pathway inhibitors. These findings demonstrated that signaling aberrations can be identified by phosphoflow cytometry, and that aberrant signaling could be normalized by small molecule drugs that perturbed specific signaling pathways. Results suggest clinical applications of such results and tests to the extent that such inhibitors are available clinically. This approach can thus identify relevant drug targets as well as drug effects in the individual patient.

## MATERIALS AND METHODS

### Patient material and ethical considerations

Buffy coats from anonymized healthy blood donors and blood samples from CLL patients were received from the Blood Centre (Oslo University Hospital) and the Department of Haematology, Oslo University Hospital, respectively, following written informed consent from all donors. Controls from the Blood Centre were drawn from a donor population where 45% were > 47 years of age. The study was approved by the Regional Committee for Medical and Health Research Ethics of South-East Norway and the research on human blood was carried out in accordance with the Declaration of Helsinki (2013). Clinical characteristics of the CLL patients included in this study are listed in Supplementary Table 1.

### Reagents and antibodies

The cytostatic drugs fludarabine, doxorubicin and vincristine, the PI3Kδ inhibitor idelalisib (CAL-101) and the STAT3 inhibitors WP1066 and niclosamide were from Selleckchem (Houston, TX, USA). The alexa Fluor 647-conjugated antibodies BLNK (pY84), Btk (pY551) & Itk (pY511), IgGkappa, Lck (pY505), MEK1 (pS298), NF-κB p65 (pS529), PLCγ2 (pY759), Rb (pS807/811), STAT1 (pY701), STAT3 (pY705), STAT5 (pY694) and STAT6 (pY641) were from BD Biosciences (Franklin Lakes, NJ, USA). Alexa Fluor 647-conjugated antibodies against Akt (pS473), Histone H3 (pS10), MAPKAPK-2 (pT334), p44/42 MAPK (pT202/Y204), NF-κB p65 (pS536), p38 MAPK (pT180/Y182), SAPK/JNK (pT183/Y185), S6-Ribosomal protein kinase (pS235/236), and SYK (pY525/526) were from Cell Signaling (Danvers, MA, USA). The anti-human surface marker PerCP-Cy5.5-conjugated CD19 was from eBioscience (San Diego, CA, USA) and anti-human PE-Cy7-conjugated CD5 was from BD Biosciences. Anti-human IgM was from Southern Biotech (Birmingham, AL, USA). The RosetteSep Human B Cell Enrichment Cocktail and Lymphoprep were from Stemcell Technologies (Cambridge, United Kingdom). BD phosphoflow Perm Buffer III and Fix Buffer I were from BD Biosciences. RPMI 1640 GlutaMAX medium, fetal calf serum (FCS) and the barcoding fluorochromes Ax488 Succinimidyl Ester, Pacific Blue Succinimidyl Ester and Pacific Orange Succinimidyl Ester were from Thermo Fisher Scientific (Waltham, MA, USA).

### Purification of B lymphocytes

B cells were purified from buffy coats by negative selection using RosetteSep Human B Cell Enrichment Cocktail (20 μl/ml blood) followed by Lymphoprep. CLL cells were isolated from whole blood by Lymphoprep according to manufacturer’s protocol. Cell samples were cryopreserved in liquid nitrogen.

### Phosphoflow experiments

The phosphoflow experiments were performed as described previously [23], with some modifications. See the following subsections.

### Stimulation and fixation

B cells from healthy donors or CLL patients were incubated in RPMI 1640 GlutaMAX medium with 1% FCS and calibrated for 10 minutes in a 37°C water bath before pre-incubation as indicated with drugs or 0,001% DMSO as vehicle control, for 20 min. An unstimulated sample was taken before the cells were stimulated with anti-IgM (10 μg/ml) for the specified time periods. The harvested samples were fixed for 10 minutes in pre-warmed BD Phosphoflow™ Fix Buffer Ι at 37°C followed by two washes with PBS.

### Fluorescent Cell Barcoding (FCB)

Fixed cells were resuspended in PBS and incubated with different concentrations of the barcoding fluorochromes Alexa Fluor 488, Pacific Orange and Pacific Blue (diluted in DMSO) in a 96-v-well plate. After staining in the dark for 20 min at room temperature, the cells were washed twice with flow wash (PBS, 10% FCS and 0.09% sodium azide), combined in one tube and permeabilized with BD Phosphoflow™ Perm Buffer ΙΙΙ pre-stored at −20°C, and stored at −80°C.

### Antibody staining and phosphoflow cytometry analysis

The permeabilized cells were washed three times with flow wash and spun for 5 min at 500g, resuspended and distributed into aliquots. The aliquots were stained with anti-CD19 surface marker conjugated with PerCP-Cy5.5 and, when indicated, with anti-CD5 conjugated to PE-Cy7, and the indicated phospho-specific antibodies conjugated with Alexa Fluor 647, before they were incubated in the dark at room temperature for 30 min. Next, the samples were washed once, resuspended with flow wash and analyzed with a BD FACSCanto ΙΙ (4-2-2) cytometer equipped with 405 nm, 488 nm and 633 nm lasers. Separately, unstimulated cells stained with Alexa Fluor 488, Pacific Orange and Pacific Blue, and compensation beads incubated with PerCP-Cy5.5- and Alexa Fluor 647-conjugated antibodies, were used for compensation. 150 000-500 000 events were recorded per sample (corresponding to 5 000-20 000 events per deconvoluted condition). Signals were calculated using the inverse hyperbolic sine (arcsinh) of the MFI (median fluorescent intensity) of phospho-signal versus isotype control, or of stimulated versus unstimulated cell populations, as described [24].

### Analysis in Cytobank

The data were analyzed in Cytobank (https://cellmass.cytobank.org/cytobank/). By plotting SSC area versus FSC area, the lymphocytes were selected. Thereafter, single cells were selected by plotting FSC height versus FSC width. B cells were gated by plotting SSC area versus anti-CD19 PerCP-Cy5.5 and the FCB was selected by plotting Alexa Fluor 488, Pacific Blue and Pacific Orange against SSC area sequentially.

### CellTiter-Glo luminescent cell viability assay and Dead Cell Apoptosis assay

CLL cells were co-cultured with CD40L+, BAFF+ and APRIL+ (ratio 1:1:1) L cells for 24 hours prior to initiation of the experiment to prevent induction of spontaneous apoptosis. The L cells were removed, and the CLL cells were incubated with the indicated drugs for 48 hours. CellTiter-Glo (Promega, Madison, WI, USA) reagent was subsequently added and luminescence was recorded after 10 min incubation at room temperature using an EnVision 2102 Multilabel Reader (PerkinElmer, Waltham, MA, USA). Alternatively, following drug incubation the cells were stained with FITC Annexin V and PI using a Dead Cell Apoptosis Kit from Thermo Fisher Scientific and analyzed with a BD FACSCanto II cytometer.

### Statistical analysis

Statistical analyses were performed with R software (https://www.r-project.org) and Prism 7 (GraphPad Software, San Diego, CA). Analysis of the phosphorylation levels for 20 phospho-proteins was carried out via hypothesis testing in order to assess the significance of the differences between the CLL groups and the control, in particular between the means in the CLL groups and the value 1 (normalized to the control). The test used was an unpaired *t*-test for each phospho-protein sample.

The sample correlation between the different phosphorylation levels in the CLL group was calculated and tested for each phospho-protein pair. The statistical test used for this aim was the Pearson’s product-moment correlation test (or Pearson’s r-test), assessing if the correlation between two variables was significantly different from 0 (positive or negative). A *p*-value less than 0.05 was considered significant for this study.

The data were clustered via hierarchical agglomerative clustering. The dissimilarity between the phospho-protein CLL samples was assessed via standard Euclidean distance, while the linkage method used was Ward’s method or average method.

## SUPPLEMENTARY MATERIALS FIGURES AND TABLES


