# Supplementary material for: Single cell profiling of phospho-protein levels in chronic lymphocytic leukemia
Source: Oncotarget. 2018 Jan 4;9(10):9273–84. doi: 10.18632/oncotarget.23949 (PMC5823631; doi:10.18632/oncotarget.23949)
Supplement: Supplementary file 1 [file oncotarget-09-9273-s001.pdf]

Single cell profiling of phospho-protein levels in chronic lymphocytic leukemia

SUPPLEMENTARY MATERIALS

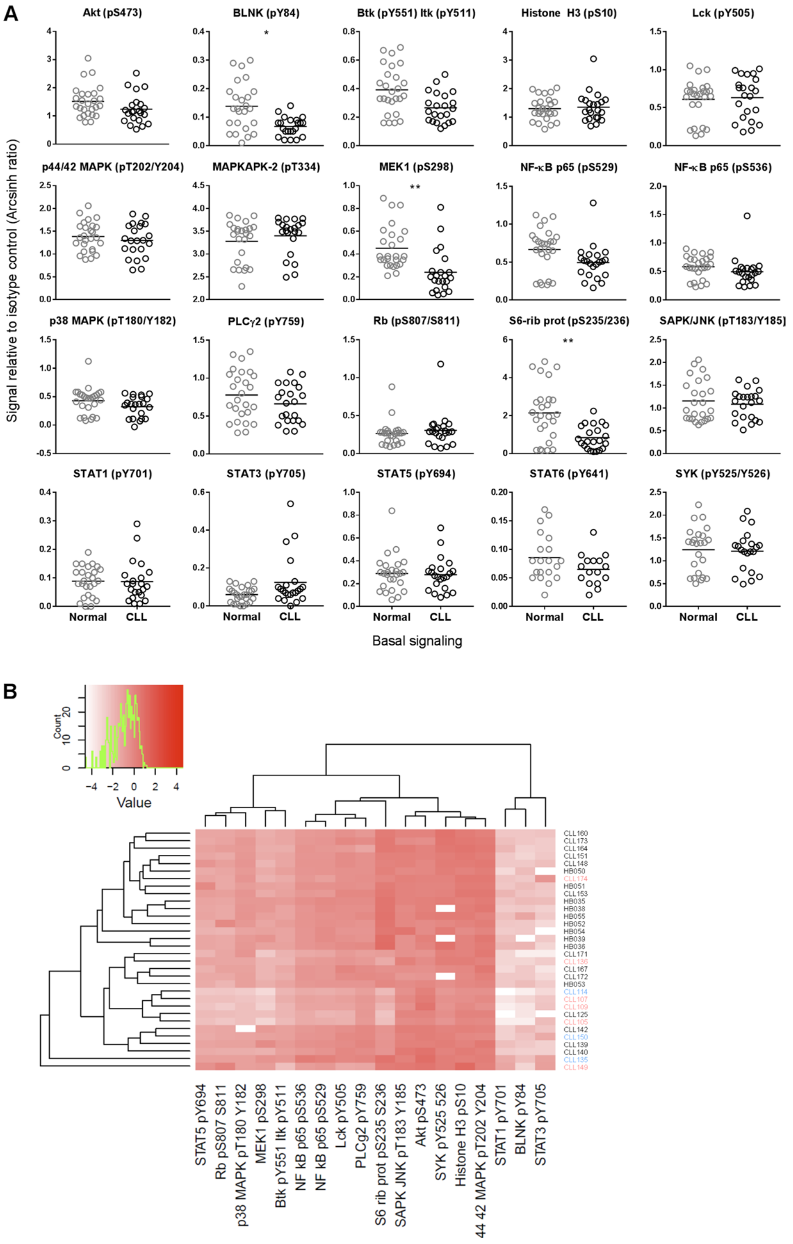

Supplementary Figure 1: Basal signaling with *p* values adjusted for multiple testing. (**A**) Basal phosphorylation levels are shown for CLL samples and normal controls used for normalization in Figure 1A. Asterisks indicate significant p-values ($^{*}p < 0.05$, $^{**}p < 0.01$) assessed by a paired t-test with correction for multiple comparisons using the Holm-Sidak's method. (**B**) Phospho-proteins and B-cell samples were grouped via hierarchical agglomerative clustering (Euclidean distance – average linkage method). STAT6 (pY641) was removed due to the presence of several missing values, as well as MAPKAPK-2 (pT334) since its high values reduced the visibility of the variability of the data. Log-transformation was applied to further emphasize the differences in the signals. Missing data are indicated in white. Patients highlighted in pink and blue refer to the STAT3 (pY705) high patients as shown in Figure 1.

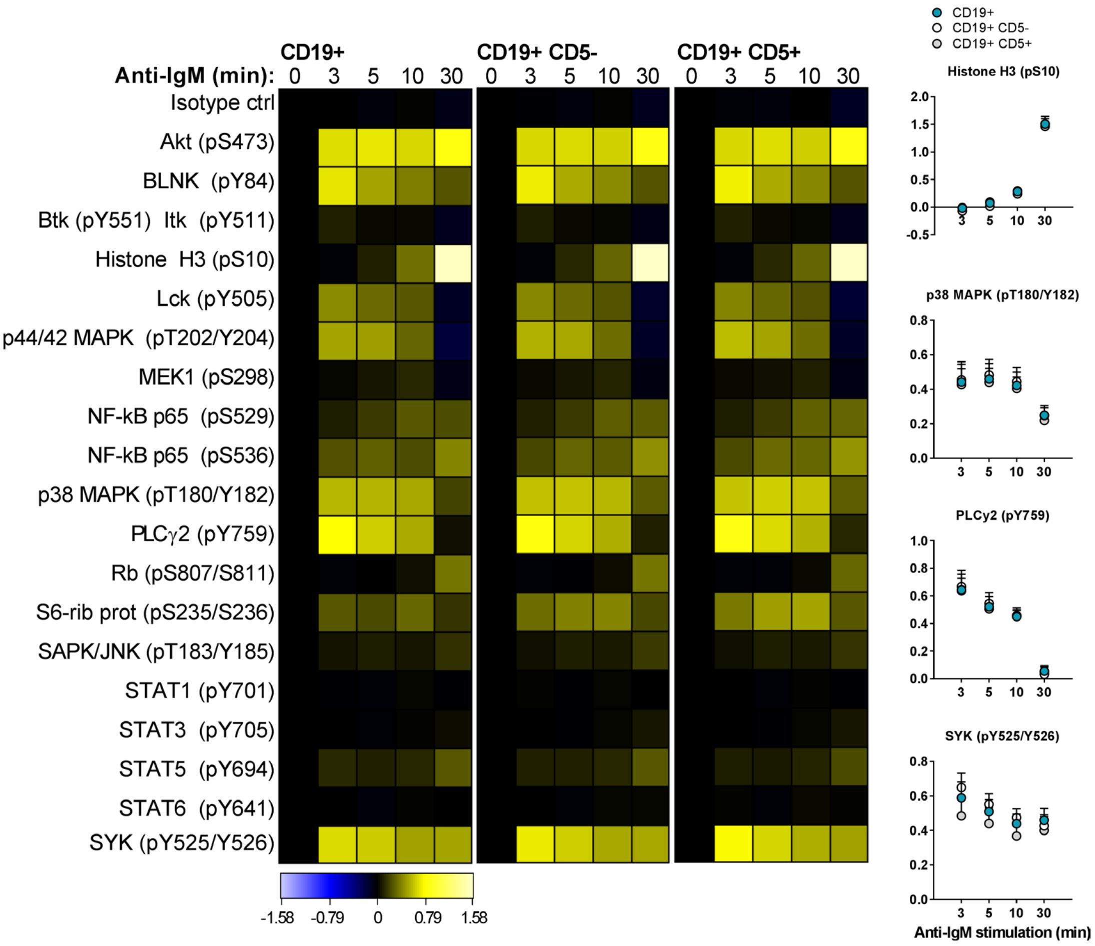

Supplementary Figure 2: Signaling patterns in CD5 subsets. B cells from healthy donors were stimulated with anti-IgM for the specified time period, then fixed and permeabilized as described in the Methods section. The cells were stained with anti-CD19 and anti-CD5 surface markers, as well as the indicated phospho-specific antibodies. The fluorescence intensity of the phospho-signals in the gated cellular fractions (CD19$^+$, CD19$^+$CD5$^-$ and CD19$^+$CD5$^+$) were measured relative to unstimulated samples and shown as arcsinh ratio. All phospho-signals are shown for one representative donor (heatmaps, left), while the average of *n* = 4 (SEM) donors are plotted for selected phospho-proteins (graphs, right).

Supplementary Table 1: Patient characteristics

PATIENT IDENTIFIER	GENDER/AGE (YEARS)	TIME FROM DIAGNOSIS TO PROCUREMENT (MONTHS)	TREATMENT PRIOR TO PROCUREMENT	BINET STAGE	IgHV-GENE MUTATIONAL STATUS (% HOMOLOGY GERMLINE)	CYTOGENETIC ABNORMALITIES
**CLL105**	F/56	150	No	A	Bi (100, 94.8)	ND
**CLL107**	M/38	138	No	A	M (92.9)	del (13q14)
**CLL109**	M/54	152	No	A	M (93.8)	ND
**CLL114**	M/55	120	No	A	M (88.2)	ND
**CLL125**	M/44	6	No	C (A at diagnosis)	Bi (97.3, 98.1)	Normal karyotype
**CLL135**	M/69	99	FCR, *R-CHOP	C (A at diagnosis)	UM (100)	Normal karyotype
**CLL136**	F/66	8	No	A	M (91.9)	del (13q14)
**CLL139**	F/61	82	No	C	M (93.2)	Normal karyotype
**CLL140**	M/64	73	No	A	M (88.7)	del (13q14)
**CLL142**	F/48	144	No	B	M (96)	del (13q14), del (17p13), TP53 mutation
**CLL148**	F/58		No	A	M (93.3)	ND
**CLL149**	M/50	28	No	A	UM (100)	ND
**CLL150**	F/60	76	FCR	B	UM (100)	del (13q14), del (17p13), TP53 mutation
**CLL151**	F/54		No	A	M (93.2)	ND
**CLL153**	M/50	24	No	A	UM (100)	TP53 wild-type
**CLL160**	M/68	103	No	A	M (86.9, 93.6)	46,XY
**CLL164**	M/73	18	No	B	UM (100)	46,XY TP53 mutation
**CLL167**	M/58	167	No	C	M (93)	del (13q14)
**CLL171**	M/53	58	FCRx2	C	UM (100)	del (13q14), del (11q22)
**CLL172**	M/68	>192	No	A	M (92.3)	ND
**CLL173**	M/41	15	No	A	UM (100)	ND
**CLL174**	M/68	120	FC	C	UM (98.8)	Normal karyotype

No; Non-treated, ND; not defined, M; mutated, UM; unmutated. Treated patients have received the following drugs marked with abbreviations: F = fludarabine; C = cyclophosphamide; R = rituximab. *CLL135 was treated with R-CHOP independently of the CLL diagnosis.
